# Extracellular vesicles in endometriosis: role and potential

**DOI:** 10.3389/fendo.2024.1365327

**Published:** 2024-04-26

**Authors:** Xinying Chu, Menghui Hou, Ying Li, Qingyue Zhang, Shuxin Wang, Jing Ma

**Affiliations:** First Teaching Hospital of Tianjin University of Traditional Chinese Medicine, National Clinical Research Center for Chinese Medicine Acupuncture and Moxibustion, Tianjin, China

**Keywords:** endometriosis, extracellular vesicles, pathogenesis, diagnosis, treatment, cell-cell communication

## Abstract

Endometriosis is a chronic inflammatory gynecological disease, which profoundly jeopardizes women’s quality of life and places a significant medical burden on society. The pathogenesis of endometriosis remains unclear, posing major clinical challenges in diagnosis and treatment. There is an urgent demand for the development of innovative non-invasive diagnostic techniques and the identification of therapeutic targets. Extracellular vesicles, recognized for transporting a diverse array of signaling molecules, have garnered extensive attention as a novel mode of intercellular communication. A burgeoning body of research indicates that extracellular vesicles play a pivotal role in the pathogenesis of endometriosis, which may provide possibility and prospect for both diagnosis and treatment. In light of this context, this article focuses on the involvement of extracellular vesicles in the pathogenesis of endometriosis, which deliver information among endometrial stromal cells, macrophages, mesenchymal stem cells, and other cells, and explores their potential applications in the diagnosis and treatment, conducing to the emergence of new strategies for clinical diagnosis and treatment.

## Introduction

Endometriosis (EMs) is a chronic inflammatory condition, characterized by the presence of abnormal tissue similar to the endometrium outside the uterine cavity, besetting approximately 10% of women of reproductive age (1). Common symptoms comprise secondary dysmenorrhea, deep dyspareunia (pain in the upper vagina during sexual intercourse), chronic pelvic pain, and infertility (2).

The pathogenesis of EMs remains uncertain and encompasses various pathogenic pathways, including retrograde menstruation, benign metastases, immune dysregulation, coelomic metaplasia, hormonal dysregulation, involvement of stem cells, and alterations in epigenetic regulation (3, 4). Laparoscopy with histopathological confirmation has been considered as the widely accepted standard for diagnostic assessment (2, 5). Nevertheless, recent evidence indicates that the prevalence of occult EMs may be as high as 39% in women who were diagnosed as negative during laparoscopy (6). The management of EMs is typically categorized into conservative approaches, hormonal therapies, and surgical treatments. However, the clinical benefits are often limited. Consequently, there is an urgent demand for comprehensive research into the pathogenesis of EMs, alongside the exploration of innovative non-invasive diagnostic techniques and the identification of effective therapeutic targets.

Cell-cell communication has been demonstrated to play a critical role in various physiological processes, including cell proliferation, development, and differentiation (7). Recent studies have suggested that extracellular vesicles (EVs) serve as a novel mechanism mediating cellular crosstalk within or among tissues. An expanding body of research indicates that EVs play a significant role in disease progression, diagnosis, and treatment (8). Simultaneously, EVs are present in body fluids and show promise for use in ‘liquid biopsy’, having been successfully separated from blood, urine, and saliva (9). There is potential for EVs to serve as a reliable, non-invasive diagnostic indicators for diagnosis of various pathological conditions (7, 10).

### Biogenesis of EVs

**Figure 1:**
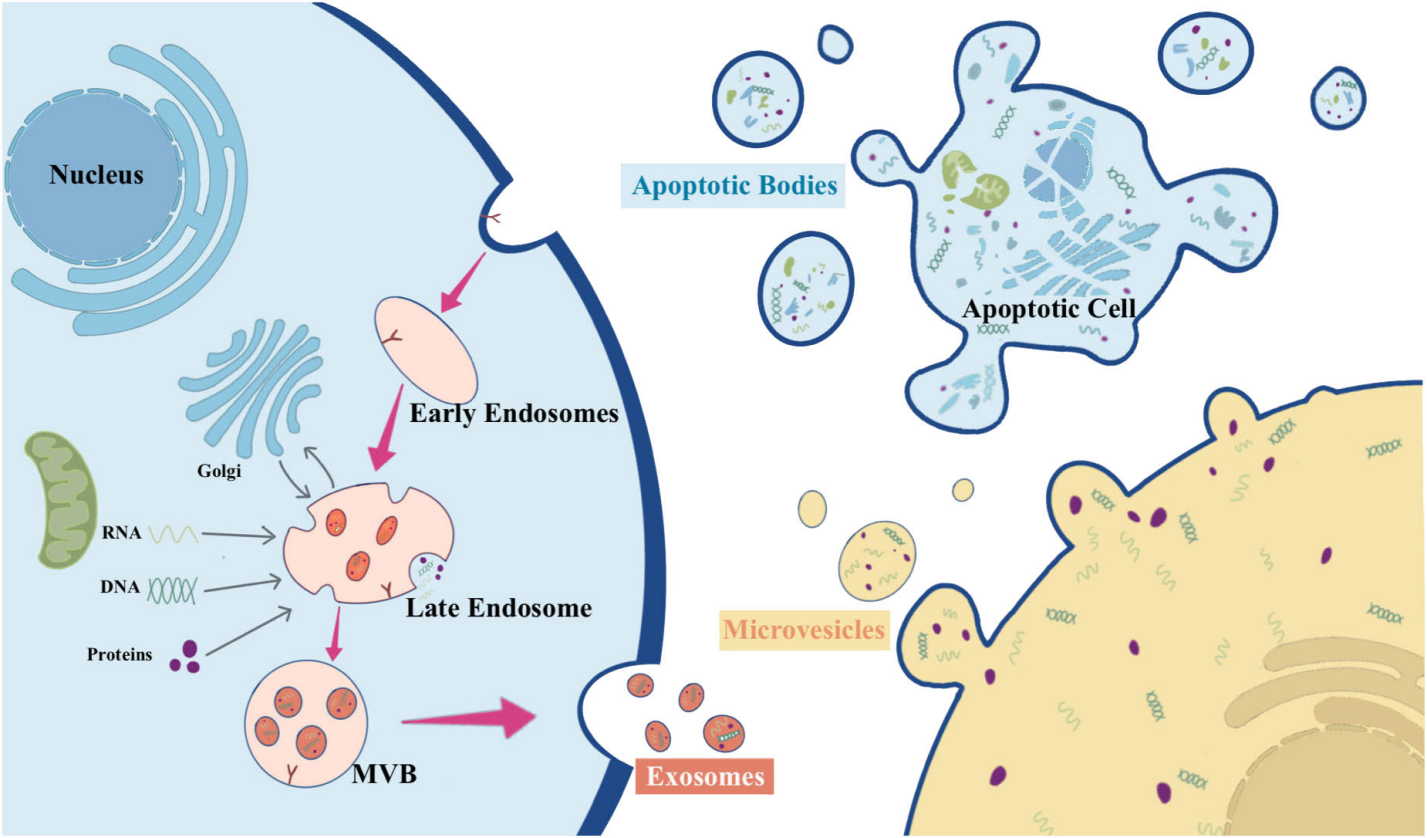
The biogenesis of different types of EVs.

EVs constitute a heterogeneous group of membrane-structured vesicles, characterized by lipid bilayer-encapsulated nanoparticles, carrying various substances such as nucleic acids, proteins, lipids, and metabolites. They are actively released by nearly all types of cells and can be found in various human body fluids, including blood, urine, saliva, and ascites. EVs function as key players in cellular crosstalk, enabling the delivery of messages over long distances or in close proximity (8, 11, 12). According to the International Society for EVs, EVs can be categorized into medium/large EVs (>200 nm) and small EVs (<200 nm) based on their physical properties (13). Based on their origin, size, biogenesis, and function, EVs are classified as exosomes, microparticles, microvesicles (MVs), apoptotic bodies, ectosomes, and oncosomes (14). **Figure 1** overviews the biogenesis of different types of EVs.

Exosomes are relatively small EVs (about 50-150 nm) released by almost all types of cells, which are produced through the endocytosis endosomal pathway (14). The biogenesis of exosomes begins with the inward budding of endosomal membranes, resulting in the formation of early endosomes. These early endosomes subsequently progress into late endosomes. Throughout this maturation process, the endosomal membrane undergoes invagination into the lumen, leading to the development of intraluminal vesicles (ILVs) that can encapsulate cytoplasmic molecules. In the course of this process, cytoplasmic proteins, nucleic acids, and lipids are sorted into ILVs. Late endosomes enriched with significant quantities of ILVs are termed multivesicular bodies (MVBs). These MVBs can either merge with lysosomes, leading to degradation, or with the plasma membrane, resulting in the release of ILVs. Once ILVs are released into the extracellular space, they are known as exosomes (14–18).

The process of MVs biogenesis is much less elucidated compared to that of exosomes. MVs typically range from about 100 to 1000 nm and can be generated in various cell types via direct outward budding and fission of the plasma membrane (15, 16, 19). The shedding process of MVs is related to the molecular rearrangement of the plasma membrane, which, in turn, is affected by protein, lipid, and Ca^2+^ levels. Elevated intracellular Ca^2+^ levels or the liberation of Ca^2+^ from the endoplasmic reticulum can trigger the activation of Ca^2+^-dependent enzymes, leading to alterations in the asymmetric phospholipid distribution of the plasma membrane and depolymerization of the actin cytoskeleton, thus promoting MVs shedding (16, 20).

Apoptotic bodies have the largest diameter, ranging from 1000 to 5000 nm. They are fragments of apoptotic cells composed of phosphatidylserine-exposing plasma membrane and cytoplasmic materials (21). In the final stage of apoptosis, cells undergo division into variable numbers of apoptotic vesicles containing a variety of cellular components, including micronuclei, chromatin remnants, cytoplasmic fractions, degraded proteins, and DNA fragments (22). The formation of apoptotic bodies involves the rupture of the cytoskeleton beneath the plasma membrane, resulting in apoptotic membrane blebbing. Disruption of phospholipid asymmetry in the plasma membrane triggers membrane blebbing, apoptotic membrane protrusion, and finally compression of nuclear vesicle fragments into a semilunar shape to release apoptotic bodies (23). Following their formation, apoptotic bodies are engulfed by phagocytes, including neutrophils, macrophages, and dendritic cells, for final degradation (24).

The biogenesis of ectosomes, typically ranging in diameter from 50 to 500 nm, diverges from that of other EVs. In ectosomes, cargo secretion takes place through the accumulation of cargo at the cytosolic surface of the plasma membrane. Subsequently, ectosomes are released into the extracellular matrix through the outward budding of the cell membrane (25). The structure of ectosomes relies on local microstructure domains assembled in the plasma membrane of the ectosomes. The biogenesis of ectosomes is attributed to the rearrangement of asymmetric membranes composed of phospholipid layers, which undergo reorganization facilitated by Ca^2+^-dependent enzymes (14).

Oncosomes are vesicles with diameters ranging from 100 to 400 nm, formed by the protrusion of the plasma membrane of tumor cells. They are typically associated with cell motility, and their release is regulated by various structural proteins that contribute to plasma membrane extrusion and rupture (26). Tumor cells spontaneously release oncosomes containing metalloproteinases with pro-immune properties, which carry unique substances delivering signals to specific target cells and serve as major regulators of the tumor microenvironment (14).

During the formation of EVs, cellular components such as DNA, RNA, proteins, and lipids are sorted into EVs and function as signaling molecules when delivered to target cells. Released EVs engage in communication with the target cell through ligand-receptor interactions, fusion, or receptor-induced endocytosis. This interaction facilitates the transfer of EVs components into the receptor cell, thereby inducing signaling pathways within the target cell. Notably, EVs are secreted in both physiologic and pathologic conditions, with contents corresponding to the state of their donor cells. This unique property endows them with the potential to be utilized as diagnostic molecules and therapeutic targets for various diseases.

### EVs are involved in mediating EMs

EVs can be released by the vast majority of cell types constituting the human body. These vesicles contain a diverse array of cell type-specific content, extensively identified as RNA, proteins, lipids, and noncoding RNAs, including microRNAs (miRNAs), long noncoding RNAs (lncRNAs), and transfer RNA-derived small RNAs (tsRNAs). tsRNAs can be categorized into two primary groups: tiRNAs (tRNA halves) and tRFs (tRNA-derived fragments). There is growing evidence of differences in the cargo carried by EVs at multiple sites in patients with EMs.

To determine the role of EVs in EMs, studies have been conducted to analyze EVs in human samples. At least 1449 mRNAs, 938 lncRNAs, 39 miRNAs, and 61 competing endogenous RNAs (ceRNAs) were identified to display differential expression patterns in EVs derived from endometrial stromal cells (ESCs) of patients without EMs compared to ESCs of patients with EMs, including ESCs from ovarian endometrioma and those from eutopic endometrium (27). Another study found 63 up-regulated and 45 down-regulated tiRNAs and tRFs in ectopic tissue EVs from EMs (28). It was also found that lncRNAs MEG8, SNHG25, LINC00293 and RP5-898J17.1 were down-regulated, while lncRNAs LINC00998, PVT1, and RP4-561L24.3 were significantly up-regulated in ectopically diseased EVs from EMs patients (29).

In addition to ectopic tissues, EVs exhibited differential miRNA expression in body fluids of EMs patients. Twenty-four miRNAs were observed to show varying enrichment levels in serum EVs of EMs patients, with miR-320a and miR-22-3p significantly upregulated (30). In peritoneal fluid EVs from EMs patients, thirteen EVs miRNAs were differentially expressed (miRNA-1908,-130b,-451a,-486-5p,-4488,-432,-342,-425,-505,-6508,-145,-365a, and -365b) and were involved in immune alterations and cell proliferation (31). Fourteen miRNAs were found to be differentially expressed in tubal fluid EVs from tubal EMs patients, with four up-regulated and 10 down-regulated miRNAs (32). MiR-210-3p, miR-20b-5p, miR-625-5p, miR-342-5p, miR-155-5p, miR-146A-5p, and miR-130b-3p were up-regulated in uterine lumen aspirates, while miR-335-3p and miR-132-5p were down-regulated (33). In leukorrhea EVs, both miR-202-3p and miR-202-5p were notably upregulated, each playing a role in advancing various diseases, including cancer (34).

The level of miRNAs can be differently regulated in EVs from different tissues of the same patients. A study found that 21 miRNAs were differentially expressed in plasma-derived EVs from EMs patients compared to healthy control plasma EVs, with miR-375, miR-27a-3p, and miR-30d-5p significantly downregulated (29). However, miR-375 and miR-30d-5p were notably downregulated in EMs ectopic endometriotic lesions compared with normal endometrium, yet were found to be upregulated when compared to matched eutopic endometrium (29). Furthermore, miR-27a-3p demonstrated upregulation in both EMs eutopic and ectopic tissues compared to control normal endometrium (29).

In addition to the previously mentioned contents, the microbiota composition of EVs in the peritoneal fluid of women with EMs was found to be significantly altered. Diversity analysis indicated notable differences between the two microbial communities at the order, family, and genus levels. In the EMs group, the levels of Acinetobacter, Pseudomonas, Streptococcus, and Enhydrobacter were significantly higher compared to the control group. Conversely, the amounts of Propionibacterium, Actinomyces, and Rothia were significantly lower in the EMs group than in the control group (35).

**Figure 2:**
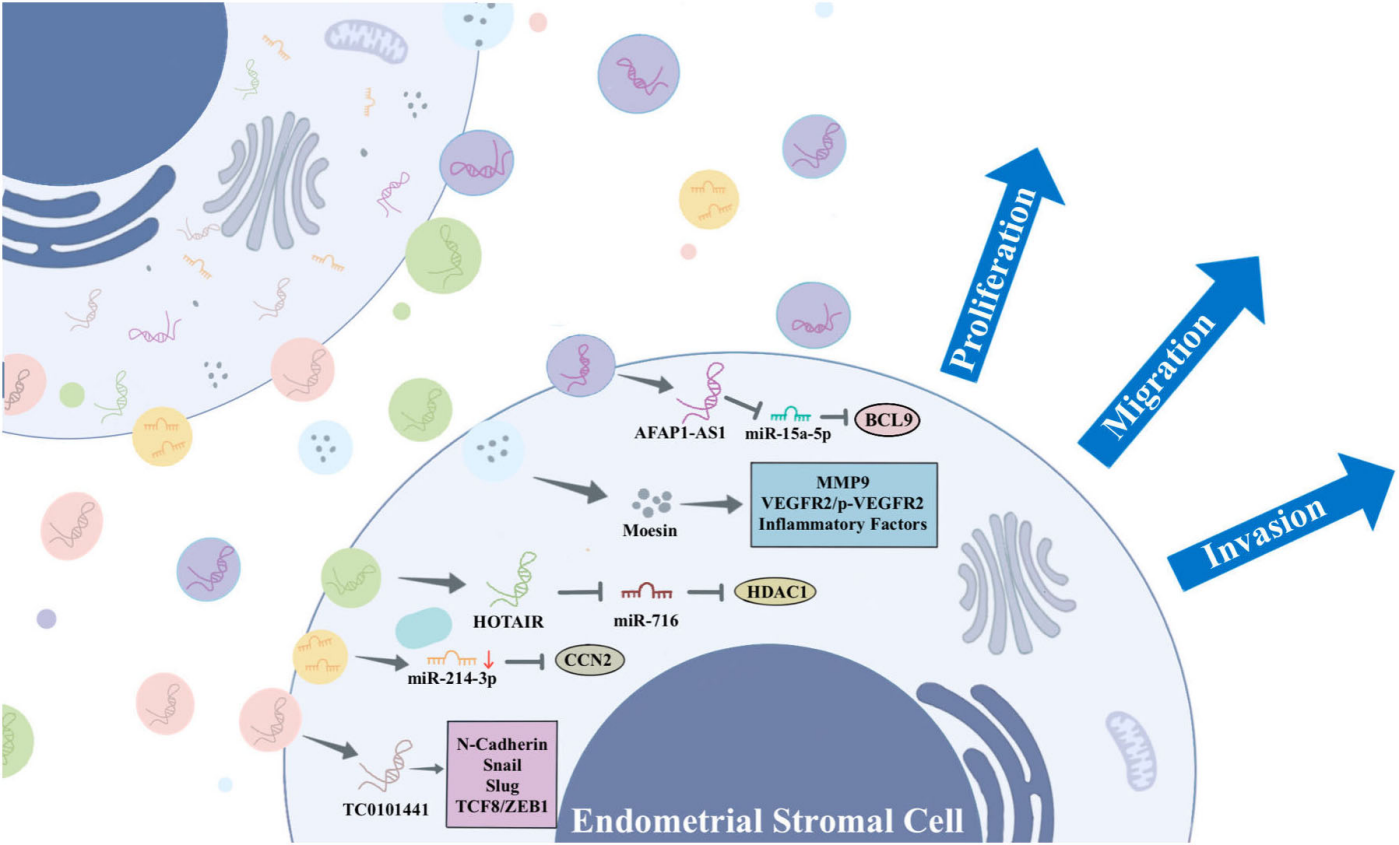
The information exchange between ESCs via EVs.

**Figure 3:**
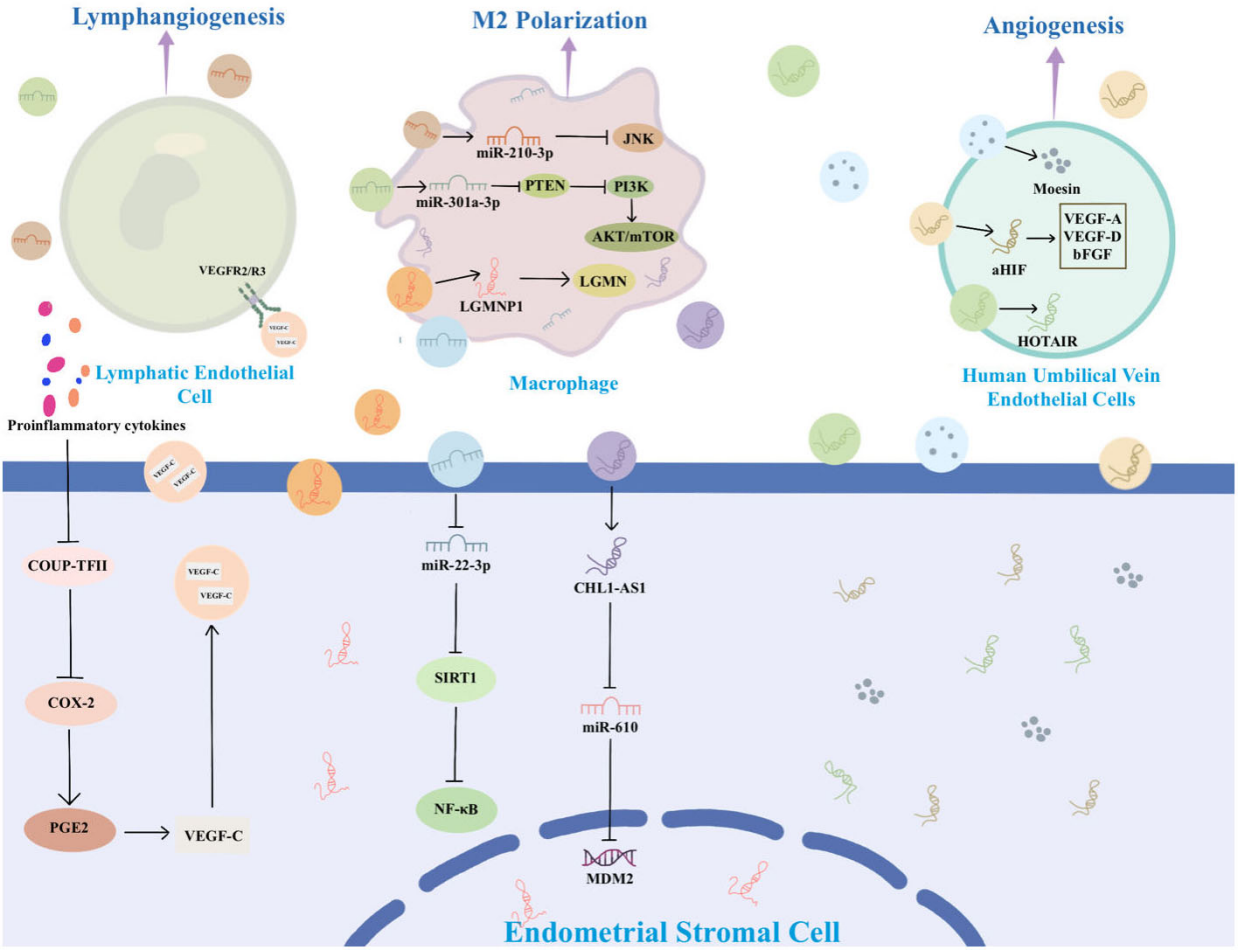
The information transfer among ESCs, macrophages, and MSCs through EVs.

An increasing body of research highlights the significance of EVs in EMs. EVs associated with EMs carry distinctive cargoes that play a role in disease pathophysiology by influencing EMs cell proliferation, immune escape, angiogenesis, and lesion invasion. EVs are released by all cells, facilitating the transfer of bioactive components from donor cells to recipient cells. This process mediates the exchange of information between cells. In the context of EMs, ESCs, macrophages, and mesenchymal stem cells (MSCs) engage in communication through EVs that carry specific substances (36–38). These EVs mediate various signaling pathways, ultimately promoting the progression of EMs. **Figure 2** summarizes the information exchange between ESCs via EVs. **Figure 3** summarizes the information transfer among ESCs, macrophages, and MSCs through EVs.

ESCs play a key role in the development of EMs by releasing EVs containing specific substances that stimulate surrounding ESCs and other recipient cells, thereby promoting angiogenesis, fibrosis, and mediating EMs. For example, Abudula et al. found that EVs derived from ESCs induced normal endometrial stromal cell migration, angiogenesis, and upregulation of inflammatory cytokine expression in ovarian cells (36). Li et al. found that EVs derived from ectopic ESCs (eESCs) promote lymphangiogenesis (39). In the peritoneal fluid, proinflammatory cytokines like interleukin (IL)-1β and tumor necrosis factor-α actively inhibit the expression of chicken ovalbumin upstream promoter-transcription factor II (COUP-TFII) in eESCs. The downregulation of COUP-TFII leads to upregulation of cyclooxygenase-2 (COX-2), which is responsible for production of prostaglandin E2 that leads to secretion of vascular endothelial growth factor (VEGF)-C (39). VEGF-C carried by EVs spreads to bind with VEGFR2/R3 on lymphatic endothelial cells to induce lymphangiogenesis toward endometriotic lesions (39). Another study suggests that EVs-mediated miR-138 promotes apoptosis through NF-κB/VEGF signaling pathway and induces inflammation via the NF-κB signaling pathway (40).

Moesin, a protein associated with cell growth, motility, and migration, exhibits up-regulation in EVs derived from eESCs. These EVs, carrying moesin, are delivered to normal ESCs. Within these recipient cells, moesin mediates the establishment of a “migration-vascularization-inflammation” loop in the ectopic environment, thereby promoting the progression of EMs (36). Sun et al. discovered that EVs play a crucial role in the progression of EMs by promoting the co-recruitment of nerves and blood vessels, inducing neurite outgrowth, and inhibiting neuronal apoptosis (41).

Long non-coding RNA (lncRNA) is a subgroup of non-coding RNAs exceeding 200 nucleotides in length. They play a regulatory role in gene expression through interactions with miRNAs, mRNAs, or proteins. Recent studies have shown that lncRNAs are essential for maintaining intracellular homeostasis and play a crucial role in the development of EMs (42–44). lncRNA HOTAIR has been reported to be associated with the progression of several diseases (45, 46). A study found that HOTAIR is overexpressed in ectopic endometrium (30). ESCs-derived EVs carrying HOTAIR propagate to peripheral ESCs, targeting the miR-761/HDAC1 axis to activate STAT3-mediated inflammation, which promotes ESCs proliferation, migration, invasion, and angiogenesis (30). LncRNA actin filament-associated protein 1-antisense RNA 1 (AFAP1-AS1) is an oncogenic lncRNA that promotes proliferation, invasion, and migration of tumour cells, and it has been demonstrated that AFAP1-AS1 plays a part in the lesions of EMs (47, 48). Wang et al. found that AFAP1-AS1 is highly expressed in eESCs, delivered to the surrounding eESCs via EVs, and up-regulated BCL9 expression by binding to miR-15a-5p, which promotes proliferation, migration, and invasion of eESCs (49). Antisense hypoxia-inducible factor (aHIF) is a known angiogenesis-related lncRNA. Qiu et al. found that aHIF is highly expressed in ectopic tissues from EMs patients, transferred via EVs and regulated VEGF-A, VEGF-D, and bFGF to promote angiogenesis (50). In addition, Qiu et al. found that the expression of lncRNA-TC0101441 was significantly up-regulated in ectopic endometrium. This lncRNA was identified within EVs derived from eESCs with heightened TC0101441 levels, exerting an impact on eESCs displaying diminished TC0101441 levels. By regulating the related metastatic proteins including N-cadherin, snail, slug, and TCF8/ZEB1, eESCs adopt more pro-metastatic behaviours, ultimately promoting migration and invasion of EMs (51).

Furthermore, miR-214-3p was observed to be significantly down-regulated in ectopic lesions and stromal cells in a mouse model of EMs. The miRNA was delivered to eESCs via EVs to inhibit the expression of connective tissue growth factor (CCN2), thereby promoting the development of EMs. CCN2 acts as a downstream target of transforming growth factor-β, and has been associated with fibrosis (52).

Macrophages are highly diverse cells found in all tissues of the body and play a crucial role in the pathophysiology of EMs. They are critical for the growth, development, angiogenesis, and neurogenesis of EMs lesions (53–55). Depending on the microenvironment, macrophages can undergo modifications and be broadly categorized into two distinct subtypes: the classically activated phenotype (M1) and the alternatively activated phenotype (M2). M1 mainly mediates tissue damage and inflammation, while M2 is mainly involved in tissue repair and anti-inflammatory processes (53, 54). Growing evidence suggests that the abdominal microenvironment in EMs patients contributes to macrophages M2 polarization to facilitate progression of EMs (56, 57). Sun et al. demonstrated that EMs-EVs can remodel the macrophage phenotype to M2 polarization and attenuate the phagocytic ability of macrophages both *in vivo* and *in vitro* (41). Jiang et al. successfully isolated EVs from the uterine cavity and observed high expression of miR-210-3p in the EVs from EMs patients. MiR-210-3p is carried and delivered to macrophages in the peritoneal cavity by EVs, inhibiting macrophage M1 polarization by suppressing the JNK signaling pathway and concurrently, promoting the migration and invasion of ESCs (33).

Legumain pseudogene 1 (LGMNP1) is a pseudogene of legumain (LGMN, also known as asparagine endopeptidase). LGMN is overexpressed in macrophages and subsequently binds to the surface of macrophages to maintain their active conformation and induce M2-like polarization (58, 59). LGMNP1 was found to upregulate the expression of LGMN, promoting glioma cancer progression (60). In a recent study, it was identified that LGMNP1 is highly expressed in ectopic lesions. LGMNP1 upregulates the expression of LGMN and directs macrophages towards an M2-like phenotype (61).

Another study found that EVs-mediated miR-301a-3p also induces macrophage towards an M2-like phenotype by regulating the phosphatidylinositol 3-kinase (PI3K)-phosphatase and tensin homolog (PTEN) axis (62). PI3K is a crucial lipid kinase involved in a multitude of cellular processes, encompassing apoptosis, autophagy, cell cycle modulation, differentiation, and cellular motility. Conversely, PTEN acts to inhibit PI3K activity (63, 64). Huang et al. found that EMs-derived EVs miR-301a-3p induces M2 macrophage polarization by up-regulating expression of PI3K and down-regulating expression of PTEN, activating the PTEN/PI3K signaling pathway (62). Contrarily, EVs derived from M1 macrophages have been observed to inhibit the migration and invasion of ESCs and hinder angiogenesis, thereby impeding the development of EMs. This effect is achieved by repolarizing macrophages from M2 to M1 phenotype (65).

The EVs containing miR-22-3p, originating from peritoneal macrophages, stimulate proliferation, migration, and invasion of eESCs through the modulation of the SIRT1/NF-κB pathway (38). An elevated expression of lncRNA CHL1-AS1 in peritoneal macrophages allows the transfer of high levels of this lncRNA to eESCs via EVs. Within ESCs, CHL1-AS1 serves as a competing endogenous RNA for miR-610, leading to the downregulation of miR-610 and subsequently upregulating the expression of MDM2 (66). MDM2 is situated within the 13–14 segment of the long arm of chromosome 12. There is growing evidence that MDM2 enhances cellular activity and promotes tumour growth (67, 68). In EMs, MDM2 positivity was higher than in normal endometrium (69). Therefore, EVs containing lncRNA CHL1-AS1 derived from peritoneal macrophages may be implicated in promoting the proliferation, migration, and invasion of ESCs, while concurrently inhibiting their apoptosis (66).

MSCs are non-hematopoietic pluripotent stem cells characterized by their capacity for self-renewal and pluripotent differentiation (70). Feng et al. discovered that EVs derived from human umbilical cord MSCs (Huc-MSCs) inhibit the expression of E-cadherin while concurrently enhancing the expression of vimentin and N-cadherin at both the mRNA and protein levels. This modulation enhances migration and invasion of endometrial glandular epithelial cells (37). Zhang et al. found that Huc-MSCs-derived EVs carry miR-100 and act on ESCs, which promote proliferation, invasion, and migration of ESCs as well as epithelial-mesenchymal transition through inhibiting heparan sulfate-glucosamine 3-sulfotransferase 2 (71).

Mast cells are granular immune cells that deposit in tissues, and there is growing evidence to support their involvement in the pathogenesis of EMs (72–75). A research collected EVs from ectopic tissues and conducted sequencing of tiRNAs and tRFs to identify the specifically expressed tRF-Leu-AAG-001 within these tissues. Further research found that tRF-Leu-AAG-001 is overexpressed in endometriotic focal mast cells and delivered to peripheral cells through EVs. This process promotes inflammation and angiogenesis in EMs (28).

In summary, intercellular communication among ESCs, eESCs, MSCs, mast cells, and other cell types plays a crucial role in promoting cell proliferation, migration, invasion, and angiogenesis. This communication is facilitated by the transport of miRNAs, lncRNAs, and proteins through EVs, ultimately regulating the development of EMs.

### EVs as diagnostic markers for EMs

The absence of specific markers presents a significant challenge in diagnosing EMs, and hampers understanding of disease mechanisms. Thus, non-invasive diagnostic biomarkers are needed. EVs, carrying disease-specific cargo, are plentiful in various body fluids and have been isolated from multiple sources in EMs patients, including blood, peritoneal fluid, uterine cavity, and leukorrhea. When compared to non-EMs patients, including those with submucosal fibroids or other benign non-endometrial lesions, variations in expression levels are evident in EVs from EM patients (**Table 1**). Significantly, EVs transport cargoes with well-maintained quantities, stability, and integrity, suggesting their potential as diagnostic biomarkers, with the expectation that they will supplant tissue diagnosis as a dependable and non-invasive diagnostic tool across numerous pathological processes (10, 33, 79–82).

**Table 1:** Source, cargo, and expression trends of EVs in patients with EMs.

EVs Sample Source	EVs Cargo	Regulation in Endometriosis	References
Peritoneal fluid	miRNA-1908、-130b、-451a、-486-5p、-4488、-432、-342、-425、-505、-6508、-145、-365a and -365b	Differentially express	(31)
	Acinetobacter, Pseudomonas, Streptococcus, and Enhydrobacter	Increase	(35)
	Propionibacterium, Actinomyces, and Rothia	Decrease	
Culture medium of ESCs	Moesin, HOTAIR, AFAP1-AS1, lncRNA aHIF, miR-214-3p, lncRNA TC0101441, miR-202-3p, miR-202-5p, miR-301a-3p, LGMNP1	Increase	(30, 34, 36, 49–52, 61, 62)
	MiR-214	Decrease	(76)
Culture medium of macrophage	miR-22-3p, lncRNA CHL1-AS1	Increase	(38, 66)
Serum	miR-22-3p, miR-320a, miR-301a-3p, VEGF-C, lncRNA aHIF, RP11-326N17.2, KLHL7-AS1, MIR548XHG, miR-214-3p, lncRNA TC0101441, LGMNP1	Increase	(39, 50–52, 61, 62, 77, 78)
	LncRNA LINC01569, RP3-399L15.2, FAM138B and CH507-513H4.6	Decrease	(78)
Culture medium of MSCs	miR-100	Increase	(71)
Leucorrhea	tRF-Leu-AAG-001, miR-202-3p, miR-202-5p	Increase	(28, 34)
Uterine aspirate fluid	miR-210-3p, miR-20b-5p, miR-625-5p, miR-342-5p, miR-155-5p, miR-146A-5p and miR-130b-3p	Increase	(33)
	miR-335-3p and miR-132-5p	Decrease	
Tubal fluid	miR-6087	Increase	(32)
	miR-6747-5p, miR-5699-5p and miR-1273f	Decrease	

Li et al. identified EVs tRF-Leu-AAG-001 expression in the leucorrhea of EMs patients, demonstrating their potential as specific indicators with high specificity and sensitivity for distinguishing EMs from other conditions (28). Additionally, elevated levels of miR-202-3p and miR-202-5p were observed within EVs isolated from both the endometrium and leucorrhea of EMs patients (34). These findings suggest the promise of miR-202-3p and miR-202-5p as potential non-invasive diagnostic biomarkers for EMs. However, further exploration is needed to determine their specific diagnostic value. Moreover, significantly elevated levels of TC0101441 and aHIF were detected in EVs isolated from the serum of EMs patients, indicating their potential diagnostic utility for EMs (50, 51).

However, only a few studies have measured the sensitivity and specificity of EVs as diagnostic markers. One study, which included 48 EMs patients and 21 controls reported serum EVs-derived VEGF-C as a biomarker with the sensitivity of 81.3% and specificity of 71.4% for EMs (39). Another study of 20 subjects and 20 controls was performed by combination of serum EVs-derived miRNA—320a and 22-3p with a sensitivity of 80% and specificity of 80% (77). Wang et al. employed 85 patients and 85 controls and reported serum EVs-derived lncRNA RP3-399L15.2 as a biomarker with a sensitivity of 67% and specificity of 98%, or a combination of the lncRNAs RP3-399L15.2 and CH507-513H4.6 with a sensitivity of 80% and specificity of 85% (78). Another study, focusing on serum EVs-derived LGMNP1 as a novel non-invasive biomarker for predicting relapse had a sensitivity of 93.3% and specificity of 75.7% (61). Although EVs are promising as diagnostic markers for EMs, the samples in these studies were limited. So further research and validation are imperative before clinical application.

The primary challenge in utilizing EVs as non-invasive biomarkers lies in the isolation and purification process. Currently, various technologies have been developed for separating different EVs, including centrifugation (ultracentrifugation, density gradient, and differential), volume exclusion chromatography, precipitation, ultrafiltration, and commercial precipitation kits. These techniques heavily rely on the biophysical and/or biochemical characteristics of EVs, such as size, density, shape, and specific surface markers. Each method comes with its own strengths and limitations, impacting the yield, purity, and quality of the isolated EVs (8, 83). Differential ultracentrifugation is undertaken as the most commonly employed “gold standard” technique for the separation of EVs. This method is characterized by its simplicity, cost-effectiveness, and minimal requirement for expertise or additional materials. However, it has the drawback of a low recovery rate and is susceptible to contamination with non-vesicular components (8, 84). In recent years, several commercial kits have been developed for the isolation and extraction of EVs. In comparison to differential ultracentrifugation, these commercial kits are simpler to operate and often yield higher amounts of EVs, demonstrating greater extraction efficiencies. However, extracts obtained using commercial kits may sometimes contain albumin impurities. The choice of the EVs separation method has a substantial impact on the subsequent analysis of the substances. Therefore, the selection of an appropriate separation method is crucial for accurate and reliable results.

### EVs as potentially therapeutic targets for EMs

The management of EMs is typically categorized into conservative, hormonal, and surgical treatments. Non-hormonal and non-surgical interventions are frequently necessary, particularly for individuals actively seeking conception. Surgical treatment is often used; however, the benefits of surgery are controversial and influenced by the surgeon, surgical technique, symptoms, disease stage, and a variety of other factors (85). Postoperative recurrence is common and the risk of recurrence increases over time. Currently, the prevention of postoperative recurrence relies heavily on suppressing menstruation, but the efficacy is limited. Hence, numerous studies have delved into new treatment methods and identified advantages of EVs, such as low immunogenicity, low toxicity, low tumorigenicity, and excellent biocompatibility. These characteristics enable EVs to traverse certain biological barriers, positioning them as potential candidates for non-cellular therapies (86, 87). Furthermore, owing to their inherent advantages in facilitating cellular communication, exhibiting high physicochemical stability, and demonstrating biocompatibility, EVs are regarded as outstanding delivery platforms for a diverse array of therapeutic agents, including proteins, miRNAs, siRNAs, drugs, and even nanomaterials (88).

Several studies have conducted preliminary investigations on the utilization of EVs in the treatment of EMs or as therapeutic targets for EMs. Davoodi et al. utilized EVs sourced from stem cells isolated from menstrual blood for therapeutic purposes, demonstrating a significant reduction in expression levels of markers associated with inflammation, proliferation, migration, and angiogenesis in endometriotic cells (89). Li et al. found that EVs derived from M1 macrophages can inhibit migration and invasion of ESCs, while promoting M2 macrophage reprogramming to M1 macrophages that inhibit EMs and block angiogenesis (65). Wu et al. found that miR-214-enriched EVs inhibit fibrosis in the EMs animal model, becoming a promising treatment for EMs (76). In addition, Zhang et al. reduced the expression of fibrosis-associated proteins (CCN2, α-SMA and collagen α1) in endometriotic lesions by intraperitoneal injection of miRNA-214-3p-enriched EVs *in vivo*. It is thought that miR-214-3p can inhibit fibrosis in EMs by targeting CCN2 (52). Wang et al. used EVs derived from human umbilical vein endothelial cells at a concentration of 120 μg/mL, resulting in a significant decrease in the proliferation and invasion of ESCs and the expression of estrogen-related genes (SF-1, ERβ, and aromatase) (90).

In addition, it was previously mentioned that exosomes from EMs decrease the proportion of M1 macrophages. This effect can be reversed by the JNK activator anisomycin, suggesting that EVs can be used as a therapeutic target for EMs (33). It was also noted that VEGF-C, carried by EVs in EMs patients, possesses the ability to promote lymphangiogenesis. Lenvatinib, a selective VEGFR2/R3 inhibitor, significantly disrupts VEGF-C signaling, thereby ameliorating this phenomenon. This suggests that EVs carrying VEGF could serve as a potential therapeutic target for the treatment of EMs (39).

Finally, existing research on potential therapeutic EVs targets is currently confined to animal models. If specific EVs phenotypes inducing EMs indeed exist, further investigation aiming to target these specific EVs to disrupt the pathogenesis of EMs may present a potential avenue for non-surgical, non-hormonal treatment regimens.

## Conclusion

EMs is a prevalent chronic inflammatory gynecological condition, often accompanied by infertility and pelvic pain. The etiology of EMs is complex, and the lesions are extensive, causing serious damage to women’s reproductive health and imposing a heavy burden on society. The pathogenesis of EMs remains elusive, lacking non-invasive diagnostic methods and more effective comprehensive treatment plans. Hence, it is crucial to seek new strategies for diagnosis and treatment. EVs have garnered widespread attention in EMs as novel biomarkers, carrying various cargoes such as RNA, proteins, and small non-coding RNAs. EVs play a communication role among cells such as ESCs, macrophages, and MSCs, triggering cell migration, angiogenesis, immune regulation, and inflammation mediation. Especially, EVs have diagnostic value for EMs and may become new therapeutic targets for this troublesome disease. Numerous studies have revealed that EVs can serve as diagnostic biomarkers for diseases. However, only a few experiments have investigated the sensitivity and specificity of using EVs as diagnostic markers, and there is significant variation in the obtained results. Some experiments have also preliminarily explored the use of EVs in treating EMs or as therapeutic targets for EMs to improve the condition, but the research is limited. Additionally, the choice of methods for isolating EVs has a substantial impact on subsequent substance analysis, but existing methods have their limitations. Therefore, the establishment of a gold standard method for the isolation and purification of EVs is also a challenge, which may restrict their clinical application. Despite the recognized importance and significance of EVs in EMs, research in this field is still in its early stages, requiring more robust evidence to support the potential clinical applications of EVs.

## Author contributions

XC: Writing – original draft, Writing – review & editing. MH: Writing – original draft, Writing – review & editing. JM: Writing – review & editing. YL: Writing – review & editing. QZ: Writing – review & editing. SW: Writing – review & editing.
